# Supplementary material for: Native lattice strain induced structural earthquake in sodium layered oxide cathodes
Source: Nat Commun. 2022 Jan 27;13:436. doi: 10.1038/s41467-022-28052-x (PMC8795208; doi:10.1038/s41467-022-28052-x)
Supplement: Supplementary file 1 — Supplementary Information [file 41467_2022_28052_MOESM1_ESM.docx]

**Supplementary Information for**

**Native Lattice Strain induced Structural Earthquake in Sodium Layered Oxide Cathodes**

Gui-Liang Xu,^1,*^ Xiang Liu,^1^ Xinwei Zhou,^2^ Chen Zhao,^1^ Inhui Hwang,^3^ Amine Daali,^1,4^ Zhenzhen Yang,^1^ Yang Ren,^3^ Cheng-Jun Sun,^3^ Zonghai Chen,^1^ Yuzi Liu^2*^ and Khalil Amine^1,5,*^

1. Chemical Sciences and Engineering Division, Argonne National Laboratory, Lemont, IL 60439, USA

2. Centre for Nanoscale Materials, Argonne National Laboratory, Lemont, IL 60439, USA

3. X-ray Science Division, Argonne National Laboratory, Lemont, IL 60439

4. University of Wisconsin-Milwaukee, 3200 North Cramer Street, Milwaukee, Wisconsin 53211, USA

5. Materials Science and Engineering, Stanford University, Stanford, CA 94305, USA

* Corresponding authors: [xug@anl.gov](mailto:xug@anl.gov) (G.-L.X.); [yuziliu@anl.gov](mailto:yuziliu@anl.gov) (Y.L.); [amine@anl.gov](mailto:amine@anl.gov) (K.A.)

**Table of Contents**

Supplementary Figures 1-12 Page 2-13

**Supplementary Figure 1|** (a) 2D contour plot of SXRD patterns during heating of Ni_0.4_Mn_0.4_Co_0.2_(OH)_2_/NaOH mixture from room temperature to 875 ^o^C. The colour represents the intensity, with red for highest and blue for lowest. The §, ☆ and * represents O3, P3 and NaOH, respectively. (b) The corresponding covariance analysis and (c) TGA analysis.

**Supplementary Figure 2|** Rietveld refinement and lattice parameter of SXRD patterns of the Ni_0.4_Mn_0.4_Co_0.2_(OH)_2_/NaOH mixture (a) before heating and (b) after heated to 500 ^o^C.

**Supplementary Figure 3|** SXRD patterns of synthesized NaNi_0.4_Mn_0.4_Co_0.2_O_2_ with quenching and slow cooling.

**

**

**Supplementary Figure 4|** C 1*s* XPS spectrum of slow-cooling sample before and after etching.

**

**

**Supplementary Figure 5|** Cycle performance of strained O3 NaNi_0.4_Mn_0.4_Co_0.2_O_2_ cathode at different conditions. Hollow and solid symbols represent discharge and charge capacities, respectively.

**Supplementary Figure 6|** (a) Ni, (b) Co and (c) Mn K-edge XANES of strained O3 NaNi_0.4_Mn_0.4_Co_0.2_O_2_ cathode at different charge/discharge states.

**Supplementary Figure 7|** (a) HAADF-STEM image and the corresponding (b) Na and (c) Ni element mapping of pristine strained O3 NaNi_0.4_Mn_0.4_Co_0.2_O_2_ cathode.

**
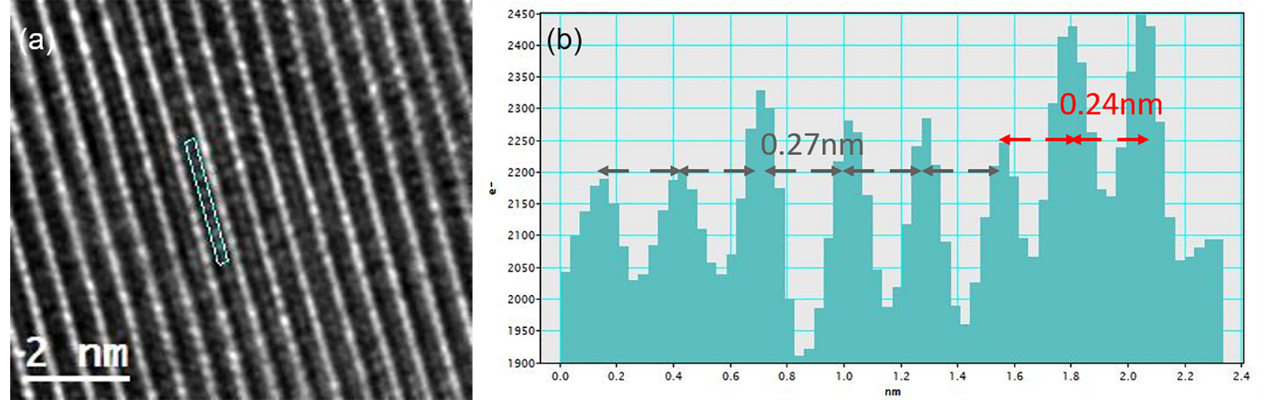
**

**Supplementary Figure 8|** (a) HRTEM image of pristine strained O3 NaNi_0.4_Mn_0.4_Co_0.2_O_2_  and (b) the corresponding line profile analysis.

**
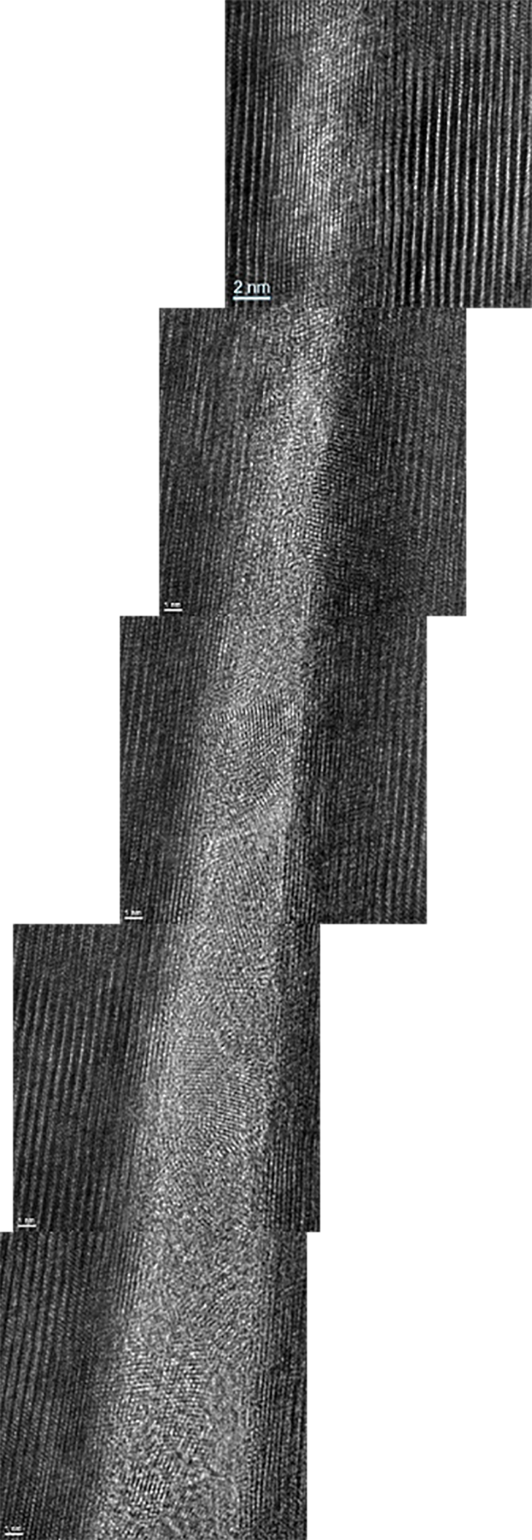
**

**Supplementary Figure 9|** HRTEM images of strained O3 NaNi_0.4_Mn_0.4_Co_0.2_O_2_  cathode after cycling within 2.0-4.4 V for 100 cycles .

**
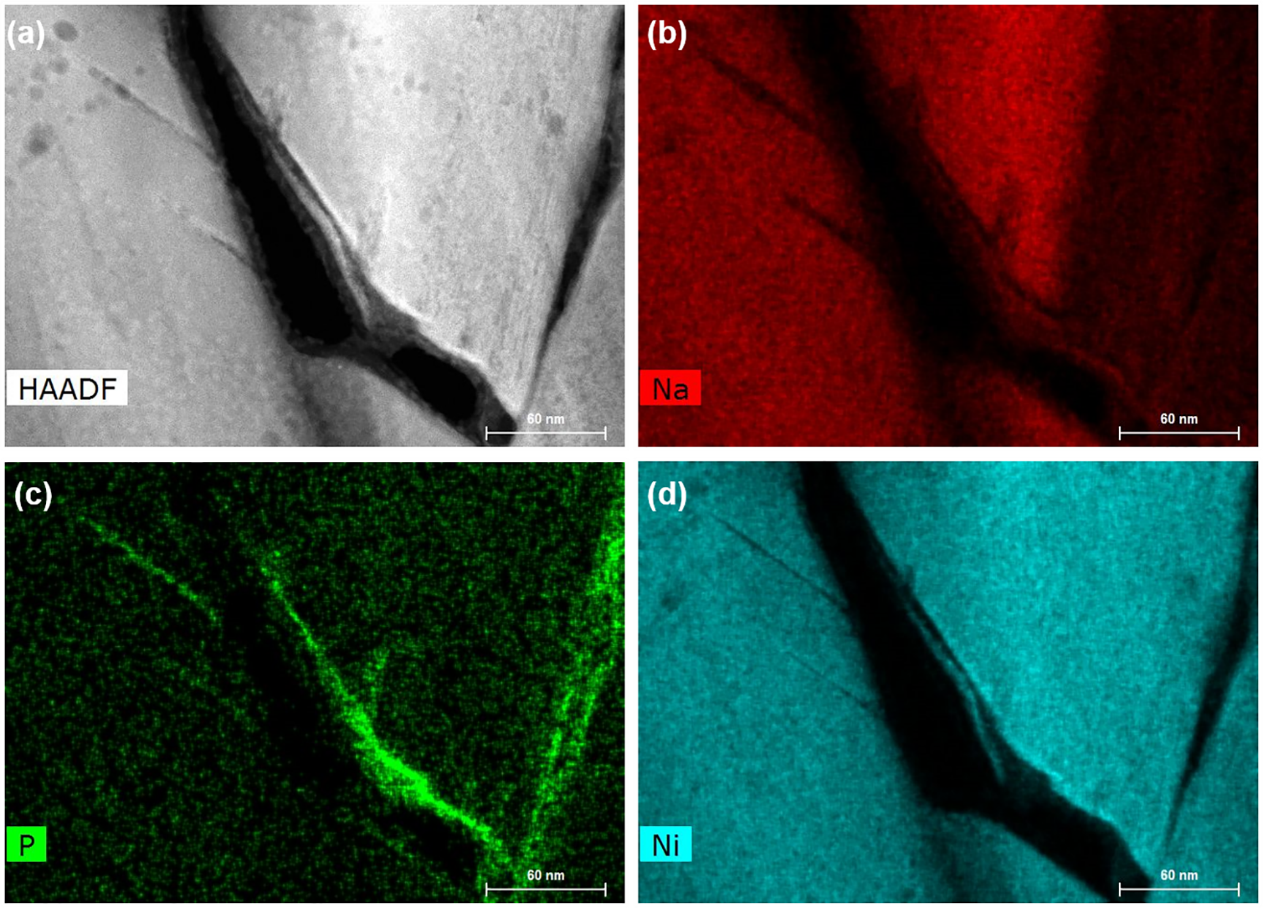
**

**Supplementary Figure 10|** (a) HAADF image and (b-d) the corresponding elemental mapping of strained O3 NaNi_0.4_Mn_0.4_Co_0.2_O_2_  cathode after cycling within 2.0-4.4 V for 100 cycles at 0.08 C.

**
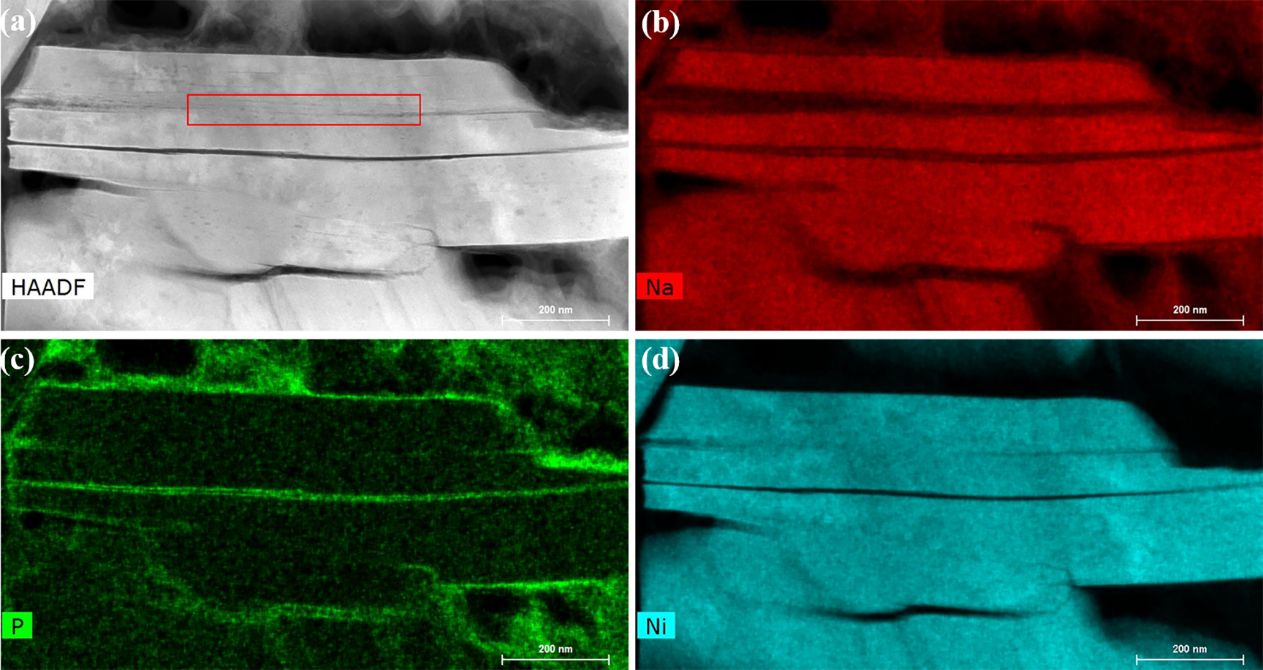
**

**Supplementary Figure 11|** (a) HAADF image and (b-d) the corresponding elemental mapping of strained O3 NaNi_0.4_Mn_0.4_Co_0.2_O_2_ cathode after cycling within 2.0-4.4 V for 100 cycles at 0.2 C and 55 ^o^C. The marked area in (a) showed no signal of Na and P, but clear signal of Ni, indicating no electrolytes penetration in this region. The results confirmed that the region is not empty and might be composed by Na-deficient rock-salt phase.

**Supplementary Figure 12|** FIB cross-sectional SEM images of O3 NaNi_0.4_Mn_0.4_Co_0.2_O_2_ cathode at various charge/discharge conditions: (a) pristine; (b) 0.08 C for 100 cycles at room temperature (RT); (c) 0.2 C for 100 cycles at 55 ^o^C; (d) 1 C for 100 cycles at RT.
